# Cultural competence in NHS hearing aid clinics: a mixed-methods case study of services for Deaf British sign language users in the UK

**DOI:** 10.1186/s12913-023-10339-4

**Published:** 2023-12-19

**Authors:** Celia Hulme, Alys Young, Katherine Rogers, Kevin J. Munro

**Affiliations:** 1 Social Research with Deaf People (SORD), School of Health Sciences, University of Manchester, Manchester, UK; 2 Centre for Deaf Studies, University of the Witwatersrand, Johannesberg, South Africa; 3 Manchester Centre for Audiology and Deafness (ManCAD), School of Health Sciences, University of Manchester, Manchester, UK; 4 Manchester University NHS Foundation Trust, Manchester Academic Health Science Centre, Manchester, UK

**Keywords:** British sign language, Hearing aids, Audiologists, Cultural competence

## Abstract

**Background:**

This study identified and explored how National Health Service (NHS) hearing aid clinics address cultural competence concerning Deaf British Sign Language (BSL) users. This was approached by (i) investigating how organisational processes meet the needs of Deaf signers from a hospital and hearing aid clinic perspective, (ii) analysing policies and guidelines to investigate if they equip practitioners to meet the needs of Deaf signers and (iii) exploring with practitioners who work in hearing aid clinics about their experiences of working with Deaf signers.

**Methods:**

This study utilised a mixed-methods multiple case study design, incorporating documentary analysis and semi-structured interviews. Interview analysis was conducted using Reflexive Thematic Analysis (RTA). The research encompassed two hearing aid clinics in separate hospitals, producing 19 documents and eight interviews (four at each site) with audiologists ensuring a representative mix of professional experience levels.

**Results:**

Four themes emerged from the integrated analysis: (1) Understanding Deaf signers; (2) Communicating with Deaf signers; (3) Barriers and Facilitators and (4) Service improvement. A noticeable gap in understanding BSL as both a language and a cultural system was apparent across various policies, strategies, training programmes and staff expertise. Over-reliance on interpreters provided a false sense of accessibility and most participants felt tentative to engage directly with Deaf signers. Positive practices observed at Sites A and B encompassed accurate identification of patients as Deaf signers, improved interpreter availability, communication methods, enhanced training and the encouragement of professional self-awareness.

**Conclusion:**

This is the first study that explores cultural competence of hearing aid clinics and its staff concerning Deaf signers in the UK. The results show both clinics require development to become an effective provider for culturally Deaf signers. Examples of how to design culturally competent practices have been provided to assist hearing aid clinics. The findings may be applicable to other underrepresented groups who are not typical users of conventional, acoustic hearing aids provided by the NHS.

**Supplementary Information:**

The online version contains supplementary material available at 10.1186/s12913-023-10339-4.

## Introduction

Cultural competence has emerged as an increasingly important consideration in professional practice in audiology services internationally. It is defined by Cross et al. [1] as ‘a set of congruent behaviours, attitudes, and policies that come together in a system, agency, or those professionals to work effectively in cross-cultural situations’ (p13). Research has addressed a range of cultural and linguistic groups and their interactions with audiology provision and service structures such as Korean speakers [2] and Spanish Hispanics speakers [3] in countries where they are minority language users. While Deaf people who use sign language are globally acknowledged as cultural-linguistic minorities [4–6] there has been a notable lack of attention given to them in the context of advancing cultural competence in audiology. This is the focus of this study.

In the UK, there are approximately 87,000 Deaf sign language users [7] who consider themselves to be part of a linguistic and cultural minority community. A growing minority of Deaf sign language users are hearing aid users [8, 9]. Conventionally marked by a capital ‘D’ to distinguish them from the larger number of people who experience their deafness and hearing loss but who do not sign. Deaf people do not perceive their deafness primarily as a biophysical inability to hear [10–12]. Instead, they view their use of language, such as BSL, as an integral aspect of their culture, which they regard as a source of gain, not loss [13]. They embrace a robust Deaf identity that is linked to language utilisation, as well as historical and cultural traditions and behavioural norms [4]. Ferguson-Coleman et al. [14] suggest that having a form of deafness does not inherently define one’s identity; instead, cultural affiliation and the use of sign language are the key factors that serve this purpose.

### Cultural competence

There is no standard definition of cultural competence. Jongen et al. [15] report a lack of consistent terminology in their systematic review of workforce cultural competence. Nevertheless, many reference Cross et al.’s [1] definition of cultural competence. Despite its popularity, Cross et al.’s definition fails to acknowledge and specify the element of language which is a significant part of cultural competence when interlocutors do not share a common language. Health care providers, including the NHS in the UK, acknowledge the significance of cultural competence in the reduction of health inequalities [16] given the disparities commonly found in health-related quality of life and health outcome amongst some minority cultural groups [17–20].

### Cultural competence and Deaf signers

Research has consistently demonstrated that Deaf signers face discrimination, barriers and inequalities in their everyday lives, including within health care services. Numerous international studies highlight the presence of barriers and challenges faced by Deaf signers when accessing healthcare services [21–25]. Not recognising Deaf people’s values and citizenships rights remains a primary concern as it is common to consider meeting needs of Deaf people arising from a disability perspective rather than from a cultural, linguistic or ethnic status [26]. Along with studies highlighting barriers with accessing healthcare, there is a growth in studies that discuss improving service provision and interventions concerning Deaf people. Persistent challenges include cultural competency training, involving Deaf people in service design and learning about and using Deaf ways of knowing (how Deaf people perceive the world and their place in it, [4] rather than just focusing on physical accessibility to services [14, 27–29].

Several articles highlight concerns pertaining to Deaf cultural competence in specific domains of practice such as health literacy, communication, interpreters, staffing, training and technology [25, 27, 28, 30–35]. However, the above literature does not evidence multiple domains of practice relating to Deaf cultural competency nor how they interact from a systems perspective.

### Cultural competence in audiology services

There is a growing body of evidence of how cultural competence is addressed when working with cultural-linguistic minorities relating to audiology. Some suggest a culturally matched link worker to provide first-hand information and support in a shared language as well as cultural brokerage between patient and service provider. This approach is exemplified by initiatives such as The Hearing and Otitis program [36], a community-based service for the Nunavik Inuit, Milpa Binna [37] and Deadly Ears [38] catering to Aboriginal and Torres Strait Islander children. Reel et al’s [3]. US based study worked with both Spanish Hispanic speakers and audiologists to develop culturally and linguistically appropriate instruction materials. Choi et al. [2] worked with US Korean older adults to adapt a community-based intervention to match the needs of Korean adult speakers. Ullarui [39] wrote a book specifically for audiologists on how to address cultural competence with their Spanish-speaking patients. In the context of Deaf signers, Cottrell et al. [40] surveyed 111 audiologists’ cultural competency by assessing their exposure, knowledge and attitudes when working with patients who are culturally Deaf American Sign Language users. The conclusion reported that audiologists’ competence is limited because of the lack of exposure to signers. Hulme et al.’s [8, 9] study on culturally Deaf signers in the UK and their experiences in hearing aid clinics identified limited Deaf awareness and cultural competence in adult hearing aid services, resulting in patient frustration and disempowerment.

Despite Hulme et al.’s [8, 9] and Cottrell et al.’s [40] recent additions to the literature, there remains a paucity of evidence relating to audiology and sign language users from a *whole service* perspective. This study explored how NHS adult hearing aid clinics in the UK address cultural competence concerning Deaf BSL users from such a whole service provider perspective incorporating data from policy, organisational documentation, professional guidance and routine hospital practices as well as directly from audiological practitioners.

## Methods

### Ethics

Ethical approval was granted by the University of Manchester Research Ethics Committee (UREC) (2020-9397-15281) and as the study was based within the NHS, ethical approval was also granted from the Health Research Authority (HRA) (20/HRA/4131).

The aim of this study was to identify and explore how hospital-based NHS adult hearing aid clinics address cultural competence in relation to Deaf BSL users.

The study objectives were to:

- Investigate the extent to which the organisational processes within adult hearing aid services were able to respond to the requirements of patients who are Deaf BSL users from a hospital setting as a whole.
- Investigate the extent to which the organisational processes within adult hearing aid services are able to respond to the requirements of patients who are Deaf BSL users from a clinic provider perspective.
- Analyse the extent to which policies and professional guidelines and training equip practitioners to meet the needs of Deaf patients in line with their professional duties and responsibilities.
- Explore with practitioners in adult hearing aid services their experiences of working with Deaf patients.

### Author positionality

The first author is a Deaf signer who uses hearing aids. She situates herself as an insider in this research, being an active member of the Deaf community and having direct experience with audiology services. The other three authors are health service researchers, one of whom is a qualified audiologist. Two of the other authors are BSL users, one of whom is Deaf, although not currently accessing audiology services.

## Design

### Patient and public involvement and engagement (PPIE)

The Deaf Experts by Experience Group (DEEG) [41], a PPIE group for health research assisted with the design phase of the study. The discussions were based on their knowledge and experiences accessing hearing aid clinics. The outputs from this discussion were a list of potential questions, an observation recording form (in the event not utilised due to COVID-19 restrictions) and suggestions for the document list to collect.

### Case selection

A multiple case study design [42] was selected for this study as it allows the researcher to investigate a phenomenon comprehensively in a real-life context. Case selection and sample size were determined by the research objectives and theoretical framework, with a preference for smaller cases to enable comprehensive exploration and richer data acquisition in line with a case study methodology approach.

Two host sites agreed to accommodate this study. The sites (Site A and Site B) were chosen for their contrasting characteristics in terms of geographical location of the hospital, population size, number of hearing aids fitted per year.

**Table 1:** Contextual description of each hearing aid clinic

Type of hospital	Population size	Hearing aids fitted per year (2019/2020)
City (Site A)	750,000	8000
Community (Site B)	155,000	3500

It is important to note that all authors have no affiliation with the sites involved in this study and data collection and analysis were exclusively conducted by the first author.

### Document data collection

Documentary analysis [43] involves examining diverse documents to extract meaningful insights through systematic review and categorisation. This method deepens understanding of a subject and complements other data collection and analysis techniques.

#### Inclusion and exclusion criteria

Only documents with current versions were included, while historical versions were excluded. No patient records were accessed either in individual or aggregated format.

#### Type of documents collected

The documents gathered for analysis were chosen through: (i) the exploration of the public-facing website of each NHS hearing aid clinic; (ii) recommendations from the PPIE group; and (iii) identification of additional operational paperwork likely to be available, such as patient referral forms and meeting minutes. The final collection encompassed internal policy and practice guidance at the hospital level, external facing policy and practice statements, internal operational practice guidance and audiology as a profession guidance.

The documentary analysis was conducted solely by the first author.

### Interviews

A qualitative semi-structured interview method was employed to investigate and explore audiology practitioners’ experiences of working in hearing aid clinics with Deaf signers.

#### Sampling

Convenience sampling was selected because of its efficiency in rapidly identifying and recruiting participants based on specific criteria. Involvement of local collaborators at each site streamlined recruitment and ensured representation across various experience levels in the hearing aid clinic.

### Selection criteria

The following inclusion criteria was used, aiming to include participants from each of the four categories: (1) registered audiologist with more than five years in role; (2) registered audiologist with less than five years in role; (3) Audiology/Healthcare assistant with more than five years in role and (4) Audiology/Healthcare assistant with less than five years in role. The rationale for these specific categories was to ensure a comprehensive representation of various levels of experience and length of exposure to Deaf signers.

Despite the request to have one person from each of the four categories per site, this was not achieved. Seven of the participants where from category one and one from category two. It is important to note that no audiology/healthcare assistants came forward for interview. Eight interviews were conducted with registered audiologists, four from each site.

### Interview data collection

The interviews were conducted online via Zoom or Microsoft Teams, and each interview averaged one hour fifteen minutes. The first author is a sign language user therefore a BSL/English interpreter was employed to interpret the interview. The interview was video recorded as BSL is a visual language and to enable the researcher to carry out translation and analysis work in the later stages. The semi-structured interviews were guided by an interview schedule (Additional File 1) which included two fictional scenarios.

### Data analysis for documents and interviews

The collected documents were manually reviewed and analysed using a documentary analysis form created by the first author. Twenty-seven documentary analysis forms were populated, but as Site A (city-based) and Site B (community-based) are separate hospitals within the same NHS Trust (the overarching organisational structure on a regional basis), they shared some of the same policies and guidelines. Therefore, there were 19 distinct documents.

The interview data were analysed using Braun and Clarke’s [44] six-step RTA approach. This framework allows for interpretations of the thematic relationships across and within cases to be made. Transcripts of the spoken English data were inputted and managed via NVIVO, a qualitative data analysis computer software package version March 2020 (R1). The original interviews including the live translation into BSL were retained during the analysis process to permit checking in BSL of any of the data in the English transcript.

Given the intended integration of the sources of data within the case study approach, the order of analysis may be influential. In this case, the interview analysis preceded the documentary analysis and themes identified in the interviews were looked for or their absence noted in the documentary analysis. Once the documentary analysis was completed, its major findings served as an additional layer of consideration in the interview analysis, with the two elements being regarded as a recursive loop. Data are not presented separately but as an integrated approach with themes identified from both documentary analysis and interviews.

## Results

### Sample

**Fig. 1:**
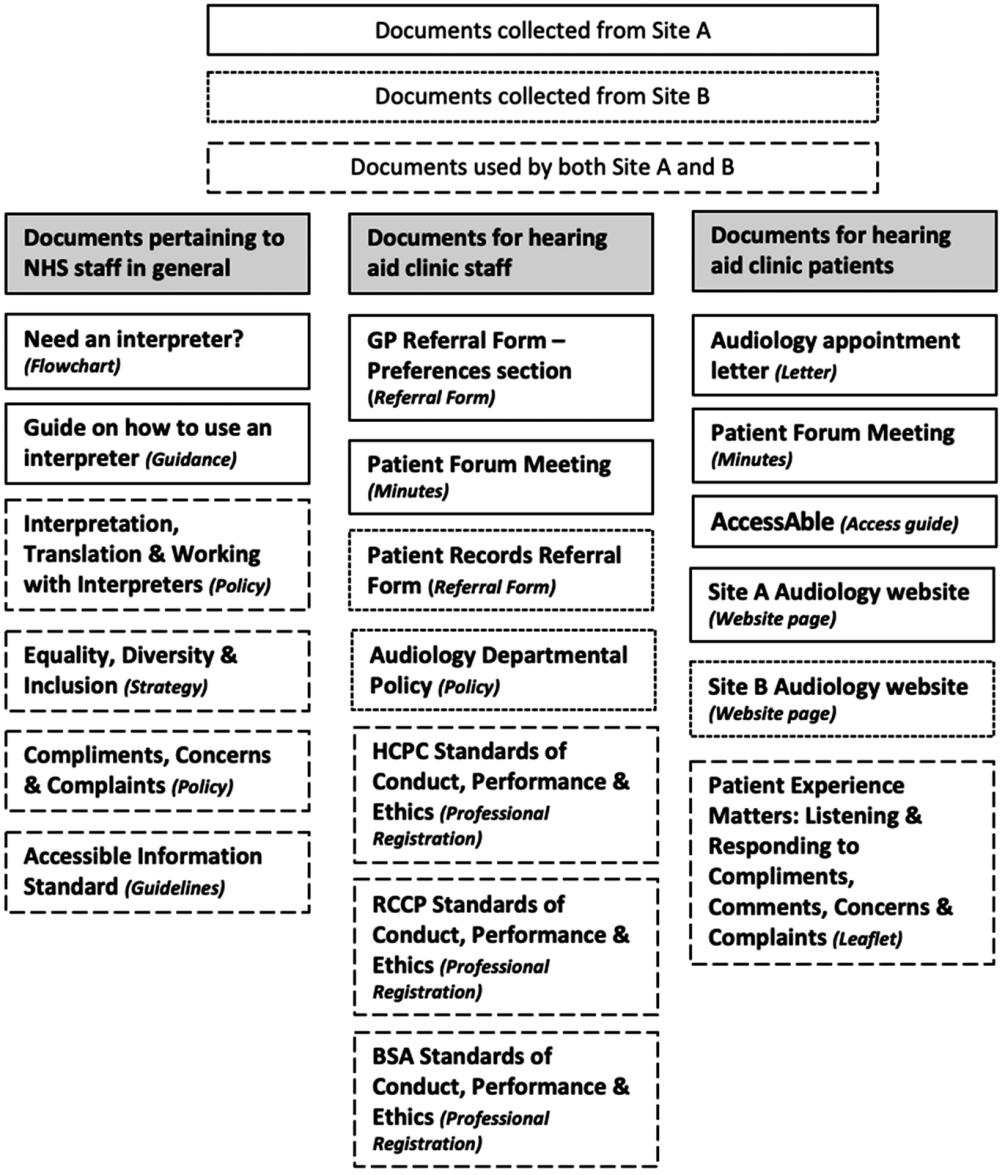
Documents collected from site A and B

Interview participant characteristics are presented in Table 2 and documents extracted for analysis at each of the two sites in Fig. 1.

**Table 2:** Site A and Site B interview participants demographics

Job title	Experience	Audiology Training	Number of Deaf signers see per year
**SITE A**			
Trainee Clinical Scientist	Less than 5 years’	MA Clinical Scientist	3 in 2 years
Audiologist	More than 5 years’	BSA (BAAT I & II)	Up to 4
Senior Audiologist	More than 5 years’	BSA (BAAT I & II)	Up to 4
Head of Audiology	More than 5 years’	BTEC and BAAT I & II	Up to 8
**SITE B**			
Audiologist	More than 5 years’	BSc in Audiology (4 years)	Up to 3
Audiologist	More than 5 years’	MA in Audiology	Up to 10
Clinical Lead	More than 5 years’	BSc in Audiology (4 years)	Up to 3
Senior Audiologist	More than 5 years’	BSA (BAAT I & II)	Up to 5

### Interview participant demographics

Participants’ experience of working as an audiologist ranged from 2 years to 42 years and most of the participants see Deaf signers fewer than five times a year. Half of the participants had completed the British Association of Audiology Technicians (BAAT) I & II training course, two completed the 4-year BSc in Audiology programme, one was qualified through the MA in Audiology, and one was a trainee on the MA Clinical Scientist course.

### Documents consulted

Documents were allocated to three different categories; (1) for NHS staff in general; (2) specific to hearing aid clinic staff and (3) for hearing aid clinic patients.

## Integrated analysis

Data from the documentary analysis and the analysis of individual interviews, although treated separately, are triangulated and incorporated in the presentation of the results in accordance with the overall case study design.

Through following the reflexive thematic analysis six step approach [44, 45] four overarching themes emerged during the integrated analysis of eight interviews and nineteen documents: (1) Understanding Deaf Signers; (2) Communicating with Deaf Signers; (3) Barriers and Facilitators and (4) Service Improvement. This inductive process was further refined by framing the results, a three level cultural competence schemata which is influenced by Cross et al’s [1], Betancourt et al’s [46] and Castillo et al’s [47] frameworks of competence. They posit three levels of cultural competence: (i) Structural Cultural Competence, (ii) Organisational Cultural Competence, and (iii) Interpersonal Cultural Competence.

1. Structural cultural competence is defined as an organisation having cultural competence at the core of all its policies, service delivery structures and decision making, which includes monitoring and evaluation. Cultural competence is not an ‘add on’ or requires special attention but runs through every aspect of an organisation’s operation.
2. Organisational cultural competence is about operational procedures as they are enacted in practice and includes the skills of the workforce and the ways in which services are delivered in practice embracing the diversity of the population it serves.
3. Interpersonal cultural competence (clinical-patient interaction) is about the ability to communicate and practice in a culturally competent way which ensures an equity of experience, outcome and standards for patients through meeting cultural needs (that may include linguistic requirements).

Each level of the cultural competence framework is not mutually exclusive and indeed theme areas have some points of intersection and overlap however together, they construct a framework to understand the complexity of cultural competence in hearing aid clinics with Deaf signers derived from the data.

### Theme 1: understanding Deaf signers

The analysis revealed that, even though most participants understood why Deaf signers use hearing aids, they did not have an adequate understanding of their culture and language.

**Structural cultural competence **- the lack of understanding of BSL as language stems from governance levels (the top). Both organisation’s policies on interpreting and translation, EDI (Equality, Diversity and Inclusion) policy and relevant departmental handbooks lacks substantial discussion on BSL, its usage and contexts. Nonetheless, BSL is partially addressed at a national level through statutory guidance, exemplified by its integration into the AIS (Accessible Information Standard) [48]. However, the focus remains primarily on practical aspects such as interpreter bookings and recording BSL users in healthcare systems. Furthermore, national regulators’ professional codes of conduct do not mandate sign language competence when working with Deaf signers as patients or service user, as seen in the Registration Council for Clinical Physiologists (RCCP), Health and Care Professions Council (HCPC) and the British Society of Audiology (BSA) standards.

**Organisational cultural competence **- Deaf awareness training was and is provided in both hospital settings, albeit somewhat inconsistently. The sporadic nature of the training has resulted in some interview participants having outdated views on Deaf culture, which resulted in three participants expressing a view that Deaf individuals involved in Deaf culture avoid using hearing aids because of potential implications for the Deaf identity within the Deaf community. This belief stems from the assumption that any technical aid (hearing aids or Cochlear Implants) poses a threat to the Deaf community. Moreover, some participants understood the concept of culture but could only cursorily apply it to Deaf people perceiving Deaf culture at a superficial level rather than integral to the person. One participant (P11) expressed this limited viewpoint, stating:

It is about community, belonging. It is the same with other cultures. People have things in common. The Deaf people have their own culture. P11

These points are highlighted because they suggest that Deaf awareness training might not reflect adequately cultural implications of being Deaf and instead focus more on hearing and communication.

**Interpersonal cultural competence** - limited access to up-to-date training on BSL and Deaf culture has caused certain participants to articulate their concerns of apprehension and unpreparedness while interacting with Deaf BSL patients within a clinical setting. In one of the fictional scenarios, the participants were asked if they would be satisfied if a leaflet in written English was given to Simon whose first language is BSL. The mixture of responses shows that some participants do not understand BSL as a separate language unconnected with English, seeing it instead as the visual version of the spoken form of English, or BSL as a simplified form of communication that can be replicated through simplified English. One participant (P09) exemplified this viewpoint stating:

If he uses a different language, we can give him a leaflet that is with his spoken language. Participant P09

However, a few participants noted that they would not give Simon a written leaflet without also arranging for someone to translate it into BSL.

Interview participants’ shared a consistent understanding regarding Deaf signers’ motivations for hearing aid use. They unanimously agreed that hearing aids were primarily used for improved access to environmental sounds rather than for speech production or comprehension. Moreover, there were no definitive stances on the desirability of Deaf signers wearing hearing aids; most acknowledged it as a matter of personal choice. This perspective contrasts with the more prevalent position of advocating strongly for the benefits of hearing aids for adults diagnosed with hearing loss/hearing disability.

### Theme 2: communicating with Deaf signers

Communication was one of the main threads throughout the analysis, where participants reported they could not confidently communicate with Deaf signers but also that they tried their best.

**Structural cultural competence** – the absence of self-evaluation practices related to communication with Deaf people was apparent in the reviewed organisational and professional documentation. This absence led to the participants’ inability to cultivate a robust ‘personal competency toolkit’ to effectively communicate with Deaf signing patients when interpreters were unavailable. While they displayed a willingness to engage, it was acknowledged by participants that their best efforts might not have met the expected standards; for example, participants tried to use fingerspelling, basic spoken language and resorting to pen and paper. However, this approach proved problematic as English was often not the patient’s preferred language and patients might lack fluency in English literacy. Using basic English without an auditory component may not be an effective strategy.

**Organisational cultural competence** – without specialised training or clear guidance on effectively communicating with Deaf signers in the absence of an interpreter, the staff lacked the necessary tools to offer proficient communication and culturally sensitive assistance. As a result, it fostered a working environment where it was acceptable that each of the participants could have a different approach and skills in communicating with Deaf signers on an ad hoc basis with no organisationally consistent standard in terms of expectations and quality for the patient.

**Interpersonal cultural competence** – when asked about the absence of interpreters, most participants tended to place the responsibility on Deaf signers to adapt their communication to suit the professionals’ abilities. This often involved simplifying spoken language, with the assumption that the patients preferred it. Some participants also emphasised creating a conductive environment for spoken communication, inadvertently disregarding the patients’ language preferences and assuming their preference for spoken language. This sentiment is echoed by the following quotes:

It would depend on their level of understanding me. I would try to keep the conversation simple and speak clearly. P15

**P11**: Those who do sign and choose not to, don’t sign because they know that staff can’t communicate with them, it is easier for them to speak.

**Interviewer**: Do you think that is alright to do?

**P11**: Yes, if they use BSL and decide not to as it is easier for them to speak, it’s no problem as long as they are comfortable to speak. If they sign, there would be communication breakdowns compared to speaking. It is easier for them. That’s the best way. P11

Participants acknowledged their heavy reliance on interpreters during appointments, in line with their NHS Trust guidelines. One participant expressed concern and unease at the prospect of a session without an interpreter, highlighting their lack of alternative communication strategies. Another participant recognised the dependence on interpreters because of the scarcity of resources available in BSL emphasising the need for more resources in BSL.

### Theme 3: barriers and facilitators

Throughout the analysis, many barriers, and facilitators to cultural competence have been accordingly distributed to the appropriate levels with the acknowledgement that they intersect in some instances and are interconnected: challenges at the structural level significantly contribute to obstacles in interpersonal interactions.

#### Barriers

**Barriers to structural competence** – all participants noted a higher likelihood of encountering Deaf signers during a drop-in or service desk sessions rather than scheduled appointments, which aligns with the general trend of patient interaction. However, they all recognised the inadequate accessibility of these routine sessions for Deaf signers, attributing it to various factors: limited staff proficiency in sign language, a lack of BSL and visual resources and the impracticality of arranging last-minute interpreters for brief drop-in sessions because of financial constraints. Notably, there was no indication of comprehensive training guidelines or specific policies for interacting with Deaf signing patients in these less structured encounters across the EDI, AIS, or audiology departments.

Analysis of the patients’ complaints leaflet revealed its unavailability in BSL, leading to initial hurdles for Deaf signers in filing complaints in their preferred language. Additionally, despite the existence of AIS guidelines in both hospitals outlining protocols for addressing communication and information requirements, their complete integration has not been achieved since their statutory establishment in 2016.

**Barriers to organisational competence** – the main challenges were predominantly linked to the disregard for making regular information and standard procedures accessible in BSL, leading to various negative consequences. Appointment letters from hearing aid clinic sites contained appointment details but lacked guidance on accessing interpreters. The absence of a clear indication of interpreter arrangements compounded the issue. Although AccessAble guides were produced for audiology patients at both sites, they were not available in BSL, rendering them inaccessible to Deaf patients. Moreover, these guides primarily focused on physical accessibility, neglecting considerations for language access. This instances highlight the systematic lack of BSL resources hindering patient comprehension of the clinical experience and impeding the promotion of patient agency. Within the NHS, patient agency involves active engagement at organisational level seeking and integrating their feedback to improve services. However, in one hearing aid clinic, despite holding patient forums, the published minutes revealed the absence of Deaf signers’ participation. The root cause of their exclusion remained unclear, as the documented evidence did not address this issue.

**Barriers to interpersonal cultural competence** – BSL courses were previously offered in the workplace but were discontinued. The justification for this decision was the perceived low benefit to the hospital/clinical unit because of the limited number of BSL-using patients. In essence, their minority status was not deemed significant enough to warrant specialised attention. However, participants identified additional obstacles to interpersonal cultural competence beyond language proficiency. These include the scarcity of BSL resources and a perceived lack of prioritisation of these patients’ needs, as highlighted by Participant 09:

We do not do it at the minute very well. Why? It is linked to resources. We could improve if we had: (1) Sign language videos – I’m sure this could happen. (2) We do our best to provide person-centred communication, but we do not extend this to sign language users. P09

#### Facilitators

**Facilitators to structural competence** – there is compelling evidence of interpreter provision, supported by clear policy and guidance (Interpreting and translation policy, how to book an interpreter and how to use them online). All participants reported using BSL interpreters to communicate with their Deaf signing patients in clinical and review appointments. Both hearing aid clinics provide suitable contacting methods for Deaf signers that are not sound-based such as text and email. Additionally, documentation from both clinics indicates the recording of Deaf signers’ preferred communication method (BSL), as mandated by AIS requirements.

**Facilitators to organisational competence** – having a policy where communication access is embedded at a structural level meant that both hearing aid clinics had access to excellent detailed guidance on booking interpreters that included which agency to use and their contact details. There was a section about training in one hearing aid clinic’s audiology handbook. Although it does not specify what training staff should receive, it did allow staff to identify their own training needs and approach the Head of Service to ask for their specific training request.

**Facilitators to interpersonal competence** – all eight participants reported using interpreters for communicating with Deaf signers. Four of the participants have BSL skills at a basic level (up to Level 1) that contribute to their communication tactics toolkit. In addition, there is evidence of Deaf awareness training provided, although inconsistently. Furthermore, two participants also reported that they do voluntary work within the Deaf community. The immersion method gave them a deeper understanding of what it is like to be a Deaf signer in everyday life and applied it to their work. Reflexivity played a vital role in cultural competence, with three participants demonstrating self-awareness linked to their signing proficiency. Notably, one participant recognised:

It is important to have an interpreter as my level of signing is not enough for an in-depth conversation or details. My signing is like broken English, it is not good enough and not appropriate. It is not fair on the person, and it is their right to have an interpreter. P11

Encouraging self-reflection fosters accountability, trustworthiness, ethical practice, personal growth and improved service delivery, thereby positively impacting patient care and satisfaction.

### Theme 4: service improvement

Participants were asked what they would improve about their services to make them more accessible to Deaf signers. There were suggestions across the three levels of cultural competence.

**Service improvement to structural competence** – all eight participants acknowledged the need for improvement in the drop-in/service desk area within their respective hearing aid clinics. One participant highlighted the challenge, stating:

We need to improve how we can work with BSL users virtually, and drop-in, this is an area of weakness. They can access the service, but the issue is communication. P13

Some participants also highlighted the necessity for exemplary models illustrating best practices, with one participant noting:

Our issue is that we do not see BSL users very often. I know it’s not right, but that is probably why there is no clear information on what we should do. They very rarely come; they are not regulars. P12

The lack of community outreach and collaboration with the Deaf community has resulted in clinical interventions being inaccessible for Deaf signers. Participants emphasized the necessity for exemplary models demonstrating how to effectively engage with the Deaf community and tailor services to meet their needs.

**Service improvement to organisational competence** – six participants indicated that BSL resources such as information in BSL need to be developed. Such examples are how hearing aids function, hearing aid maintenance and simple communication phrases if no interpreter is present. Additionally, there were calls for an increased number of staff proficient in sign language, rather than relying solely on the current limited number. Recommendations for community outreach initiatives were also put forth as a means of enhancing service provision. Training consistently emerged as a prevalent theme across all proficiency levels, with four participants specifically highlighting the need for improved training. One participant aptly noted:

Someone should tell us that there is a gap, then they would provide us with training on how to improve that gap. We don’t know what Deaf people want and what they want us to change, once we know we can receive training to improve that gap. P12

**Service improvement to interpersonal competence** – this was fundamentally a potential consequence of improvement at structural and organisational levels with key markers being regular training that provides up-to-date information on the Deaf community and its current views. The improvement of individuals’ personal communication tactics toolkit was far less evident as a recognised priority.

## Discussion

This study aimed to explore cultural competence of audiology clinic staff with respect to Deaf BSL users with a three-tier cultural competency framework [1, 46, 47] adapted for the first time to this setting and patient group. The primary findings indicate that the structural and organisational efforts to promote cultural competence overlooked Deaf sign language users as a distinct cultural group. The study has also demonstrated that policies, training, and practices that do focus explicitly on Deaf sign language users rarely understand cultural competence as anything more than an issue of language and interpreters. In other words, cultural competency is seen in the narrow frame of disability access, rather than in the more fundamentally complex framework of identification, recognition and respect of needs and requirements arising from cultural identity, cultural preferences, strengths and common practices [49, 50].

### Structural cultural competence: it’s not just about interpreters

Linguistic access, understood as interpreter provision, is the primary focus of policy documents and practice guidance concerning cultural competency. Whilst it must be applauded that there is interpreting provision and clear guidance on its necessity, the problem is that fulfilling these conditions has come to be synonymous with the service addressing the entirety of the needs of Deaf patients. As Napier and Skinner [51] found in a study of police use of BSL interpreters with Deaf signers, the reactive component of linguistic access was fulfilled (interpreters provided when needed), but the proactive component of linguistic access was barely touched. For example, the broader communication and cultural needs of Deaf signers were not embedded in policies and guidance. This finding is replicated in this context of audiology clinical services.

De Meulder and Haualand [52] argue that having interpreters as default access could mask the need for other language-concordant requirements of Deaf signers, something they refer to as the ‘illusion of inclusion’ [53]. In this study, the over-reliance on interpreters generated a false sense of accessibility, which meant other areas were overlooked, such as drop-in services where no interpreter is provided and the absence of audiology-related information in BSL. The deficiency of strategic foresight is very evident here. Nevertheless, for cultural competence to function effectively, there needs to be recognition and understanding that Deaf signers are a cultural-lingual minority group [4, 12]. Changing from Deaf awareness training to BSL awareness training would be an important indicator. The UK government’s recent passing of the BSL Act 2022 [54] may well act as a catalyst for the more specific understanding and strategic inclusion of Deaf people as a cultural-linguistic group in strategic policies where this has been lacking.

### Organisational cultural competence – better in collaboration with Deaf people

As identified above, the only culturally competent component regarding Deaf signers is the provision of BSL interpreters. Such emphasis restricts the vision of what a culturally competent provision would look like in all its aspects. For example, the scarcity of patient resources in BSL is prominent throughout the findings. Numerous studies have demonstrated how crucial it is to have information on BSL as health literacy amongst Deaf people is very poor [34, 55–57]. Hearing aid clinics could collaborate with Deaf organisations that work with the Deaf community to produce culturally appropriate BSL resources. Kornak [35] who produced cancer information in sign language and Palese et al. [30] a culturally appropriate pain tool assessment are excellent examples of successful cultural adaptation partnerships with the Deaf community.

Culturally validated and appropriate clinical practices are also an area for potential development. For example, the translation and validation of standardised patient measures in BSL, some with culturally appropriate clinical cut-offs [58] are now well established in other branches of medicine and health in the UK [59, 60], including health-related quality of life monitoring through the EQ-5D-5 L [61]. However, patient-led measures of service outcome, satisfaction, and health for audiology patients in BSL are yet to be considered and a long way from consistent implementation. However, this is the case also in the wider health and social care landscape where patient satisfaction measures in BSL are lacking [62, 63] and BSL accessible means of patient complaints and feedback are lacking despite being specifically recommended in formal guidelines in the NHS such as the AIS [48] and Interpreting and Translation Services in the NHS [64].

### Interpersonal cultural competence – it’s more than your signing ability

One of the significant consequences of becoming reliant on interpreters, whether attitudinally or in practice, is that clinical staff have few communication tactics outside of an interpreter-mediated interaction. Their understanding of potential patient engagement skills they could use, regardless of their fluency in BSL, was limited. For example, expressions of empathy, rapport building, and active ‘listening’ are patient-related skills that participants seemed not to recognise were still required, even though they could not sign. As Betancourt et al. [46] have argued with addressing racial/ethnic disparities in health and health care, regular education and training would enable hearing aid clinic staff to be equipped with knowledge, tools, and skills to communicate effectively, where ‘communicate’ implies more than using an interpreter.

Unfortunately, the scarce training provided in both hearing aid clinics did not fulfil the needs of Deaf signers as most participants did not fully understand BSL as a language and what the implications might be for patient care. It is important to note that some undergraduate audiology training programmes, introduced in 2002, generally provided Deaf awareness training with optional BSL courses when it was a four-year programme. However, when it became a three-year programme in 2012, the curriculum was reduced, and this affected Deaf awareness training. This has given those on the new courses less opportunity to learn Deaf awareness until they are employed and their workplace provides the training. Furthermore, the demographic data in Table 1 shows that, on average, each audiologist sees up to five Deaf signers per year, which does not fully expose them to the real-world experiences of Deaf signers and they only see them in a clinical context. Cottrell et al. [40] carried out a study in the US to assess audiologists’ exposure, knowledge and attitudes toward individuals within the Deaf culture. They found that most audiologists use interpreters, have basic signing skills and have low exposure to Deaf signers. Their findings further corroborate the findings from this study, meaning the low exposure to Deaf signers, reliance on interpreters and lack of direct communication with Deaf signers is a worldwide issue.

Two recent studies by Hulme et al. [8, 9] identified that Deaf signers are less likely to use interpreters or use BSL when attending hearing aid clinics. This means that the number of Deaf signers is probably higher than the clinics’ estimation. If hearing aid clinics were more competent in making BSL visible and providing opportunities for Deaf signers to communicate in BSL (first point of contact and reception), more Deaf signers would likely make themselves known to the service. In addition, Panning et al. [65] assessed audiologists for the need to have clinically relevant sign language. They found that whilst most attended a sign language course, they did not use most of the signs as they were not relevant. Therefore, making sign language and awareness courses specific to Deaf signers [27, 28] would make participants think ‘beyond the ear’ and look at situations from a Deaf perspective. This culturally competent training should be made mandatory and provided regularly.

## Implications for practice

Table 3 combines Cross et al.’s [1], Betancourt et al.’s [46] and Castillo et al.’s [47] competence framework principles to show markers and examples of how cultural competence could be carried out in hearing aid clinics concerning Deaf signers based on the findings and discussion from this study. Implementing the recommendations will enhance health outcomes, provide a better quality of care and improve patient satisfaction.

**Table 3:** Markers and examples of how cultural competency can be carried out in adult hearing aid clinics

**Structural**	- Principles established for culturally competent service concerning Deaf signers to be embedded in policies, strategies, and guidelines - Systems in place for cultural competence provision - Exemplars of good practice are provided - Regular monitoring and evaluating of the culturally competent priorities
**Organisational**	- Audiology department handbook to include a cultural competence plan concerning Deaf signers - Service provision is provided by a culturally competent workforce - Collaborative partnerships with Deaf organisations/ community to produce culturally appropriate materials and develop patient engagement for service feedback - Develop a long-term training plan for new and existing practitioners - Run patient feedback forums specifically for Deaf signers and patient satisfaction questionnaires to be available in BSL
**Interpersonal**	- Delivery of BSL Awareness and clinically relevant sign language courses which are provided and run by Deaf signers - To have a culturally competence toolkit to draw upon when communicating with Deaf signers (BSL information and culturally adapted tools) - To do outreach work with the Deaf community - To engage in regular reflexive practice (individually, as a team and with the Deaf community)

## Limitations

The potential limitation of this study is that the findings are based on two hearing aid clinics that belong to the same NHS Trust, which means they share the same policies and strategies and guidance documents. Additionally, the small number of sites involved poses a challenge to generalisability, as it is uncertain whether other hearing aid clinics and audiological staff would yield similar outcomes to those observed in this study. Furthermore, the initial intention was to interview four participants with five years or less experience. However, this study only recruited one person within this category and there were no healthcare assistants involved. It would have been helpful to compare those with less experience against those with experience in dealing with Deaf signers. The lead author’s identity as a Deaf BSL user was also a potential limitation. Professionals might have been reluctant to fully express their thoughts for fear of offending or be more likely on their guard with their remarks in a way they might not be with a hearing fellow professional.

## Conclusion

This is the first study that explores the cultural competence of hearing aid clinics and their staff concerning Deaf signers in the UK. Deaf signers need to be more considered in the service design of hearing aid clinics from a cultural-linguistic perspective, and there needs to be less reliance on interpreters to build a competent toolkit to communicate with Deaf signers. Examples of how to design culturally competent practices have been provided to assist hearing aid clinics. The findings may be applicable to other underrepresented groups and cultural minorities who are not typical users of acoustic hearing aids.

### Electronic supplementary material

Below is the link to the electronic supplementary material.

Supplementary Material 1: Interview Schedule

## Data Availability

The datasets used and analysed in this study are available from the corresponding author upon reasonable request.

## Abbreviations

AIS: Accessible Information Standard
BAAT: British Association of Audiology Technicians
BSA: British Society of Audiology
BSL: British Sign Language
DEEG: Deaf Experts by Experience Group
EDI: Equality, Diversity and Inclusion
HCPC: Health and Care Professions Council
IAPT: Improving Access to Psychological Therapies
NHS: National Health Service
PPIE: Patient and Public Involvement and Engagement
RCCP: The Registration Council for Clinical Physiologists
