# Supplementary material for: Cultural competence in NHS hearing aid clinics: a mixed-methods case study of services for Deaf British sign language users in the UK
Source: BMC Health Serv Res. 2023 Dec 19;23:1440. doi: 10.1186/s12913-023-10339-4 (PMC10731837; doi:10.1186/s12913-023-10339-4)
Supplement: Supplementary file 1 — Supplementary Material 1: Interview Schedule [file 12913_2023_10339_MOESM1_ESM.pdf]

INTERVIEW SCHEDULE

Cultural Competency: NHS Adult Hearing Aid Services and Deaf British Sign Language (BSL) Users

The interview style is loosely based on a semi-structured interview approach. This is a prompt sheet where there are a few main themes for discussion with the participant. The 'interview schedule' is purely the basis for initiating and guiding the conversation. It is not aimed to be prescriptive and the participant is to take the lead during the conversation.

- Welcome
    - Introduction
    - Purpose of the interview using the Participant Information Sheet
    - Explain why they have been chosen
    - Explain how the information will be used and shared after the interview process
    - Answer any questions
    - Consent

- Key questions (questions may be further probed if responses are vague, observe non-verbal clues that indicates further exploration).

    GENERAL

    - Briefly describe what you do in this hearing aid clinic (assess, diagnose, treat, drop-in, reception)
    - How long have you been working here?
    - What professional qualifications do you have?
    - Do you follow a Code of Conduct? If so, whose? (BAA, BSHA, hospital)

    EXPERIENCE OF WORKING WITH BSL USERS

    - Have you come across patients who communicate in sign language?
    - If so, how many a year do you see?
    - How do you communicate with them? (use sign language – what qualifications? use interpreters, they talk with you, use pen/paper, don't communicate)
    - If you don't see any, why do you think that is? (they don't come, don't use BSL in clinics)

    CULTURAL COMPETENCY KNOWLEDGE

    - Have you received Deaf awareness training? If so, what was the length of training? (delivered by whom? What did you learn?)
    - Do you know the reasons BSL users wear hearing aids?

- How would you respond to the following scenarios?

  - Scenario 1

    Grace is a 35-year-old female who is a Deaf sign language user who wears two hearing aids. She has booked an appointment for a hearing aid review and a British Sign Language (BSL)/English interpreter will be present. However, at the last minute, the interpreter does not turn up, how do you communicate with Grace? What would you do? (prompts – why make that choice?

  - Scenario 2

    Simon is a 24-year-old male who is a Deaf sign language user who wears one hearing aid. An interpreter is not booked for the new hearing aid fitting appointment and he prefers not to use one for this appointment. English is not his first language and he has low literacy (reading and writing skills). You provide him with a written leaflet about his new hearing aid. Do you think this is appropriate? (Prompts: If yes, why? If no, what alternatives? Are you able to provide them?)

OVERALL

- Do you think you and the hearing aid clinic provide an accessible service for Deaf BSL users? (if yes, describe what makes it accessible, if no – what could be improved)

- Closing questions
  - Is there anything that we have not covered that you would like to share or comment on?
  - Summarise main points
- Next steps
  - Briefly explain what will do next (analysis, feedback)
  - Thank you for taking part
